# The influence of vitamin D supplementation and strength training on health biomarkers and chromosomal damage in community-dwelling older adults

**DOI:** 10.1016/j.redox.2023.102640

**Published:** 2023-02-21

**Authors:** Agnes Draxler, Bernhard Franzke, Sanja Kelecevic, Alexander Maier, Jelena Pantic, Simon Srienc, Katharina Cellnigg, Scoris-Marian Solomon, Carina Zötsch, Rudolf Aschauer, Sandra Unterberger, Patrick A. Zöhrer, Laura Bragagna, Eva-Maria Strasser, Barbara Wessner, Karl-Heinz Wagner

**Affiliations:** a Department of Nutritional Sciences, University of Vienna, Austria; b Research Platform Active Ageing, University of Vienna, Austria; c Centre for Sport Science and University Sports, University of Vienna, Austria; d Vienna Doctoral School for Pharmaceutical, Nutritional and Sport Sciences (PhaNuSpo), University of Vienna, Josef Holaubek-Platz 2, 1090, Vienna, Austria; e Karl Landsteiner Institute for Remobilization and Functional Health/Institute for Physical Medicine and Rehabilitation, Kaiser Franz Joseph Hospital, Social Medical Center South, Vienna, Austria

**Keywords:** DNA instability, Micronuclei frequency, Chromosome abnormality, Cholecalciferol, Vitamin D supplementation, Elderly, Resistance exercise

## Abstract

Older adults lack of proper physical activity which is often accompanied by vitamin D deficiency. Those factors are known to contribute to health issues in the later years of life. The main goal of this intervention study was to investigate the effect of different vitamin D supplementation strategies for 4 weeks solely or combined with a 10-week strength training program on chromosomal stability in peripheral blood mononuclear cells in community-dwelling older people. One hundred women and men (65–85 years) received either vitamin D3 daily (800 IU), a monthly dose (50.000 IU) or placebo for 17 weeks. All groups received 400 mg calcium daily. The fitness status of the study participants was measured using the 30- second chair stand test, the handgrip strength test and the 6-min walk test. The cytokinesis block micronucleus cytome (CBMN) assay was applied to analyze chromosomal anomalies, including cytotoxic and genotoxic parameters. Changes in antioxidant markers were measured in plasma.

Walking distance and chair stand performance improved significantly. Increased levels of the parameters of the CBMN assay were detected for all intervention groups at study end. At baseline micronuclei (MNi) frequency correlated significantly with BMI in both sexes (females: r = 0.369, p = 0.034; males: r = 0.265, p = 0.035), but not with vitamin D serum levels. In females, body fat (r = 0.372, p < 0.001) and functional parameter using the 30-s chair stand test (r = 0.311, p = 0.002) correlated significantly with MNi frequency. Interestingly, not vitamin D supplementation but 10 weeks of resistance training increased MNi frequency indicating elevated chromosomal instability and also adverse effects on antioxidant markers including glutathione and FRAP were detected in the group of community-dwelling older adults.

## 1 Introduction

The number of older people continues to grow worldwide. Accordingly, the prevalence of age-related chronic diseases such as cardiovascular diseases, type 2 diabetes, and age-related cancer but also sarcopenia and the time linked to poor health is expected to increase [[1], [2], [3], [4]]. Aging is associated with a decline in physiological and physical function resulting in reduced physical activity all of which are driving factors to the onset of chronic diseases and physical impairment [5,6]. Older adults are often deficient in micronutrients such as folate, vitamin B12, zinc or vitamin D [[6], [7], [8], [9]]. Specifically, vitamin D insufficiency has been shown to have detrimental effects on the immune system, inflammatory and healing processes of fractured bones and also cardiovascular health beyond other musculoskeletal effects [10]. Vitamin D might exert also protective effects against reactive oxygen species that can otherwise trigger DNA damage and chromosomal instabilities [7,11,12]. This can not only lead to cellular death, such as necrosis or apoptosis, but also induce inflammation leading to organic malfunctions that might elevate cancer risk [13]. Micronuclei frequency (MNi) is a firmly-established biomarker for biomonitoring the extent of DNA damage and genomic instability which is widely used to evaluate cancer risk [13,14]. Aging correlates with higher levels of micronuclear frequencies (MNi) in blood lymphocytes, which can be measured using the cytokinesis block micronucleus assay (CBMN assay) [9,15,16]. The increased presence of MNi indicates elevated levels of DNA damage and mutations that play a vital role in cancer development [17]. Recent metanalyses revealed that higher MNi frequency is linked to blood and colorectal cancer [19,20]. Besides old age and the sex of a person, nutritional status and physical activity are linked to the extent of chromosomal stabilities [9,20,21]. Franzke et al., observed that vitamin B12 and folic acid might reduce chromosomal instability in older individuals [22]. Nucleoplasmic bridges (NPB) can be detected during cell division as an anaphase bridge between the two nuclei in binucleated cells. They are part of dicentric chromosomes and are covered by a nuclear membrane suggesting direct evidence for chromosomal instability in binucleated cells [23]. Further, nuclear buds (NBD) are micronuclei-like structures that are attached to parts of the nucleus and might have different mechanistic origin than MNi [23]. Chromosomal stability can be maintained by preventive strategies such as improvements in the diet and physical activity including resistance training. These lifestyle changes improve cellular resistance by inducing adaptations following challenging events, such as strenuous exercise or environmental exposure (cold, heat) which lead to acutely increased oxidative stress [9,16]. Vitamin D is assumed to be a key regulator of oxidative stress and inflammatory parameters [24,25]. For example, low vitamin D levels are associated with chronic oxidative stress that might also play a role in the onset of chromosomal damage [25,26]. Oxidative stress can often be detected through changes of plasma biomarkers, such as increased malondialdehyde (MDA) levels [27], reduced total antioxidant capacity (FRAP) and decreased ratios of reduced to oxidized glutathione (GSH:GSSG ratios) [[27], [28], [29]]. Nevertheless, data regarding vitamin D supplementation in older adults is not conclusive. Vitamin D deficiency in elderly people is associated with increased hospitalizations [30]. A recent study indicate that older community-dwelling adults display associations of vitamin D deficiency with reduced muscle strength and physical performance. For example, low muscle strength was found in 21.6% of participants with vitamin D serum levels of more than 50 nmol/L. However, 40.4% of the participants with lowest serum 25(OH)D levels under 30 nmol/L showed diminished muscle strength. Additionally, physical performance in study subjects with lowest vitamin D levels showed highest prevalence of impaired performance abilities with approx. 25.2%, but only 7.9% of the participants with sufficient vitamin D serum levels had a decreased physical performance [31]. Some studies suggest that vitamin D might exert a protective role against frailty in community-dwelling older adults [32]. Vitamin D supplementation might also improve balance and gait functions in older vitamin D deficient individuals [33]. One study showed improvements in chronic low-grade inflammatory markers in sarcopenic patients when receiving protein-based vitamin D supplementation over a period of 13 weeks [34]. A recently published metanalysis found that vitamin D supplementation could reduce the total cancer mortality risk [35]. A systematic review and metanalyses found a high prevalence of vitamin D insufficiency in patients with breast cancer and colorectal cancer [36], while other studies could not confirm these results [37,38]. One major aim of the European NutriAging Vitamin D project was to investigate the effects of different vitamin D regimen combined with a guided resistance training on all marker of the CBMN assay in healthy, community-dwelling vitamin D insufficient older adults.

## 2 Material and methods

The data presented in this paper are part of the NutriAging Vitamin D study, which is an interdisciplinary project of the University of Vienna and the Comenius University in Bratislava. The study was approved by the ethics committee of the University of Vienna (Reference number: 00390) and registered at clinicaltrials.gov (NCT04341818).

### 2.1 Study population

One hundred community-dwelling women and men (aged 65–85 years) have been recruited via local newspapers, online, radio and articles in between January 2019 and March 2019. The subjects were assessed based on inclusion and exclusion criteria published previously [18]. In short, the participants were mentally fit (Mini Mental State Examination >23) to participate in this study and they were free of physical impairments and diseases that would contraindicate to take part within the trial. Further, the subjects did not undertake resistance training for more than once per week in the last six months before starting the study. All participants displayed serum vitamin D levels less than 30 ng/mL at baseline and their health status has been investigated by study physicians. All study participants signed a written informed consent according to the Declaration of Helsinki.

### 2.2 Study design

**Fig. 1:**
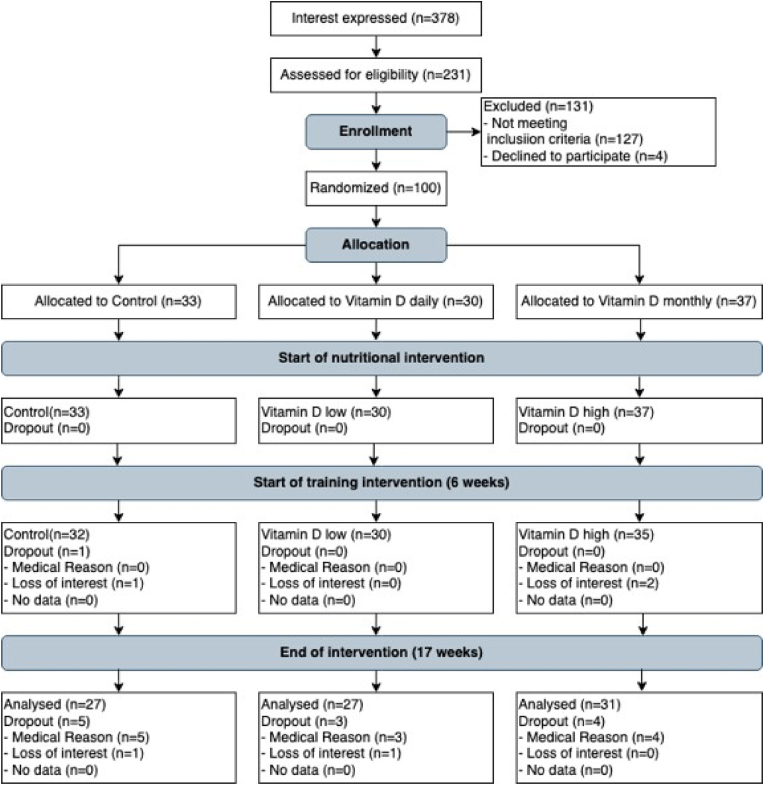
Flow chart of the intervention study according to Aschauer et al. [18].

The study design of this randomized placebo-controlled double-blind trial was described in greater detail in Aschauer et al., 2022 [18] (Fig. 1). All study participants who met the inclusion criteria were randomly allocated into three intervention groups, either the control group (CON), the vitamin D daily (VDD) or the vitamin D monthly group (VDM). The study period was divided into two phases. In the first phase, the study participants received either vitamin D supplements or placebo over a period of four weeks. In the second phase of the study, the supplementation was combined with strength training for additional 10 weeks in all study groups. All groups received in addition 400 mg calcium daily over the whole study period as intervention studies and metanalyses indicate a reduced risk for bone fractures and falls in people at an advanced age but also in prevention of osteoporosis in postmenopausal women when combining calcium together with vitamin D supplementation [[39], [40], [41]]. Blood samples were collected, and physical performance and functions were performed at baseline (T1), after 4 weeks (T2) and at the end of the intervention after 17 weeks (T3). Additionally, Vitamin D serum levels have been monitored at the 3rd, the 9th and the 13th week throughout the study.

### 2.3 Supplementation regimens of the study groups

All participants (n = 100) were divided into three groups: Control (CON), Vitamin D daily (VDD) or Vitamin D monthly (VDM) groups. The CON group consumed placebo consisting of 400 mg calcium (Mamisch GmbH Prorenal, Ludwigshafen, Germany) daily. The VDD group received 400 mg calcium 800 IU of vitamin D3 (Vitactiv Natural Nutrition, FeelGood Shop BV, Venlo, The Netherlands) daily. Participants of the VDM group were provided with 400 mg calcium daily and 50.000 IU vitamin D3 once every four weeks. The supplements were placed into 28-day pill boxes and handed out to the study participants in order to be able to consume their daily intake supplementations at home. To ensure that all supplements have been consumed, the returned pill boxes were controlled for remaining pills by study staff. Additionally, all study participants were advised to consume the vitamin D and calcium supplements daily, always at the same time, together with a meal.

### 2.4 Resistance training

The first part of this study covered a period of 4 weeks of vitamin D supplementation only. In the following second part of the study all study intervention groups received an additional supervised resistance training guided by a sports scientist two times per week for 55–75 min. A detailed training program is elaborated in Aschauer et al., [18]. Briefly, the resistance training protocol was conducted according to the guidelines of the American College of Sports Medicine and the American Heart Association for elderly people [42]. After a familiarization period to the resistance exercises in the initial two weeks of the training, future training intensity was evaluated, by testing the individual 5 repetition maximum. Training intensity and quality were tightly controlled by sports scientists and adapted according to the individual training progress. The training groups consisted of no more than five study subjects at once at each session. Each workout started with a 5-min warm up using a cross trainer, treadmill or stationary bike. Following this, the participants underwent the resistance training consisting of eight exercises. Each training session was finished with stretching exercises.

### 2.5 Thirty-second chair stand test

In order to conduct the 30-s chair stand test, a standardized seat of 43 cm height was used which was attached to a force plate. Briefly, within 30 s the study subjects had to complete as many repetitions as possible of switching between sitting and a full standing position with arms crossed over their chest. Further, if the participant had finished more than 50% of their final attempt within the last second, it was regarded as a valid repetition [43].

### 2.6 Handgrip strength test

A dynamometer was used to measure isometric handgrip strength (kg). The participants were sitting during the measurements and the maximal isometric contraction was assessed within 4–5 s using the JAMAR compatible handgrip dynamometer. Two consecutive trials for each hand were conducted and for each participant and the best results were recorded. For the analyses, handgrip strength of the dominant arm was used [44].

### 2.7 Thirty-second arm curl test

The arm curl test was conducted as published by Rikli and Jones [45]. First, the participants were familiarized to the test without using weights. During the testing, dumbbells were used and both arms were tested (males: 3.6 kg dumbbell, females: 2.3 kg dumbbell). The study subjects were sitting and completed as many as possible arm curls in the period of 30s. Only correctly performed arm curls were counted and included into the analyses.

### 2.8 Six-minute walk test

To assess aerobic endurance, the study participants had to walk along a 30-m shuttle track in the fastest 6-min walk test pace as far as possible for 6 min. Additionally, the participants were also allowed to reduce their 6-min walk test speed during the testing and if needed to take short breaks. The 6-min walk test was carried out and runs were repeated. The time with the faster performance in seconds was considered as valid result [45].

### 2.9 Timed up and go

Mobility of the participants was assessed using the timed up and go test in which the study subjects had to sit on a chair followed by standing up as fast as possible. Next, the participants were 6-min walk test 3 m in order to turn around a cone, followed by a return to the chair and sitting. All subjects were familiarized with the movements before the actual testing took place [46,47].

### 2.10 Cytokinesis block micronucleus cytome assay

After overnight fasting, 16 mL of venous blood samples have been collected from the participants in the morning using EDTA tubes (Greiner Bio-One, Kremsmunster Austria). For the isolation of peripheral blood mononuclear cells (PBMCs), Ficoll density graduation was applied using Ficoll separation tubes (Greiner Bio-One). The protocol of Fenech et al. [15] was applied to conduct the cytokinesis block micronucleus cytome (CBMN) assay as described previously [[48], [49], [50], [51]]. For stimulating the PBMCs into mitotic cell division, a concentration of 1 × 10^6^ cells/mL in culture medium were treated with phytohaemagglutinine (PHA-Sigma- Aldrich). The PBMC samples were incubated at 37 °C and 5% CO_2_ for 72 h. After 44 h of incubation cytochalasin B (Sigma Aldrich, Vienna, Austria) was added in order to stop cell division. In the next step, the cells were loaded on microscope slides using a Cytospin (Thermofisher Scientific) and stained using Diff-Quick (Medion Diagnostics, Dudingen, Switzerland). A bright field microscope (1000- fold magnification; Olympus, Vienna, Austria) was used for counting the cells. It should be noted that duplicates of each PBMC sample were produced twice resulting in four slides per sample. In total 2000 cells per participant were counted. In order to reduce scorer bias and to decrease experimental variation, 500 cells of each slide were scored by two different scorers that were also recalibrating scoring accuracy regularly with standardized slides. For evaluating chromosomal stability of the PBMCs for each participant, MNi frequency was counted. Further, not only nucleoplasmic buds and bridges were scored but also the number of necrotic and apoptotic cells in total of 2000 binucleated cells. Additionally, the nuclear division index was calculated according to Fenech et al., to be able to assess mitogenic response and cytostatic effects of the PBMCs. The scoring was conducted by three different scorers to be able to manage counting the high number of CBMN microscopic slides of the study.

### 2.11 Reduced and oxidized glutathione (GSH and GSSG)

In order to analyze reduced glutathione (GSH) and oxidized glutathione (GSSG) and the GSH:GSSG ratio as a marker for oxidative stress, a modified method published earlier using N-ethylmaleimide and o-phthalaldehyde [52,53] was applied. Fluorometric analyses were carried out using BMG FLUOstar OPTIMA Microplate Reader (BMG LABTECH GmbH) and all samples were determined in triplicates and external standards of GSH and GSSG were used [52].

### 2.12 Measurement of malondialdehyde (MDA)

MDA plasma levels were assessed in duplicates as published earlier [54]. First, samples were heated to 100 °C for 60 min and subsequently neutralized using methanol/NaOH. After centrifugation at 3000 rpm for 3 min, MDA levels were determined using high-performance liquid chromatography (HPLC). Excitation was measured at 532 nm and emission at 563 nm (LaChrom Merck Hitachi Chromatography System, HPLC column: 125 × 4mm, 5 μm, Merck).

### 2.13 Analysis of ferric reducing ability potential (FRAP)

To assess the antioxidant capacity of the plasma, the ferric reducing ability potential assay (FRAP) was used as published by Benzie and Strain [28]. The absorbance of the serum samples was measured in triplicates at 593 nm using the FLUOstar OPTIMA Microplate Reader (BMG LAB-TECH GmbH). Additionally, trolox was measured as the control antioxidant standard for each assay. Trolox equivalents (μg/L) were used to express the results.

### 2.14 Measurement of anthropometric parameters

Anthropometric parameters have been assessed as described in Unterberger et al. [55]. Bioelectrical impedance analyses (BIA) was used to measure body composition, including body fat (kg), body cell mass (kg), lean body mass as well as total body water. The measurement of bioelectrical characteristics of the human body such as resistance and reactance were used to calculate impedance [56,57]. Waist to Hip ratio (WHR) was calculated by dividing the waist measurement (cm) with hip measurement (cm). Body mass index (BMI) was calculated for each participant using the formula: BMI = weight/(height)^2^ [kg/m^2^].

### 2.15 Measurement of blood parameters and vitamin D

Following an overnight fasting, venous blood drawing took place between 6:30 and 9:00 in the morning. Two EDTA-stabilized blood collection tubes containing 8 mL each (Vacuette, Greiner bio-one, Kremsmünster, Austria) were kept for 30 min at room temperature and then transported to the Laboratories of the Department of Nutritional Sciences of the University of Vienna for harvesting PBMCs that were further subjected to the CBMN assay as described above. Further, Serum Sep Clot Activator collection tubes (Vacuette, Greiner bio-one, Kremsmünster, Austria) were filled with 8 mL venous blood that was used to assess vitamin D3 serum levels. After 30 min at room temperature, the clotted blood was centrifuged at 3000 rpm for 10 min at room temperature. In order to analyze 25(OH)D2 and 25(OH)D3, the Access 25(OH) Vitamin D Total assay (Beckman Coulter Austria, Vienna) was used. Right after blood drawing, the blood collection tubes were transported into a routine medical laboratory (Dr, Claudia Vidotto study lab GMBH, 1230 Vienna) to measure vitamin D serum levels, plasma hs-C-reactive protein (hs-CRP), hs-Troponin and general blood parameters including erythrocytes, hematocrit and hemoglobin status.

### 2.16 Statistics

For statistical analyses IBM SPSS Statistics 27 was used. In order to evaluate normality of the data, all variables have been tested using the Shapiro-Wilk-test and by visually inspecting boxplots and QQ-plots. To analyze correlations between variables related to CBMN parameters at baseline, Pearson correlation and Spearman rank correlation was used. Since some parameters were not normally distributed, non-parametric tests were opted for to assess the intervention effects. The main differences between the time points were measured using the Friedman-Test. To measure baseline group differences, Mann-Whitney *U* test and Kruskal-Wallis-H was used. Bonferroni correction (p < 0.005) was considered when the Friedman test with a p-value below 0.05 revealed significant differences. The percentage of variation between the timepoints T1 and T2, T2 and T3 as well as T1and T3 were calculated. The differences (Δ) between phase 1 (T2-T1) and phase 2 (T3-T2) were calculated and Wilcoxon test was used to evaluate potential differences between the phases (T2-T1 and T3-T2).

## 3 Results

### 3.1 Demographic characteristics

One-hundred participants were included into the NutriAging Vitamin D study, consisting of 67% males and 33% females at baseline. Thirty-three study participants were allocated to the CON group (23 males/10 females), 30 participants to the VDD group (20 males/10 females) and 37 subjects started the intervention in the VDM group (24 males/13 females). The mean age of the participants was 70.6 ± 4.5 years. The mean age for men was 70.3 ± 4.7 years and the mean age of the women was 71.2 ± 4.7 years. Overall, the study subjects had a BMI of 27.3 ± 4.7 kg/m^2^. Males had a mean BMI of 27.1 kg/m^2^, whereas females displayed a mean BMI of 27.7 kg/m^2^. Regarding the CBMN assay, data of 97 subjects could be generated at baseline (64 males/33 females).

### 3.2 Sex-specific differences at baseline

Women showed differences regarding the total number of cells with MNi, apoptotic cells and also the nuclear division index was significantly different compared to men (Table 1). In terms of body composition, muscle mass was significantly lower in females compared to males. Conversely, body fat was significantly higher in women. Functional parameters at baseline, revealed that women needed significantly more time in the timed-up and go test and covered a shorter distance within the 6-min- walk test and the 30-s chair stand test (Table 1). Oxidative stress parameters including MDA, GSH, GSSG, the GSH:GSSG ratio as well as FRAP were not different between males and females (Table 1). In males, blood testing revealed lower hs-CRP levels in the group with lower MNi frequencies at baseline (p < 0.05, Table 2). In contrast, female participants displayed no significant differences in body composition, functional parameters or blood parameters between the groups with lower vs higher MNi frequencies. However, in females the number of nuclear buds were significantly reduced in the group with higher numbers of MNi frequency at baseline (Table 1 Supplementary).

**Table 1:** Baseline characteristics of participants.

Parameter	All	Men	Women	p-value
Subjects	n = 100	n = 67	n = 33	
	Mean ± SD	Mean ± SD	Mean ± SD	
Age [years]	70.6 ± 4.5	70.3 ± 4.5	71.3 ± 4.7	0.397
Vitamin D serum level [ng/mL]	22.8 ± 5.5	22.4 ± 5.8	23.7 ± 4.8	0.174
**CBMN parameter**	n = 97	n = 64	n = 33	
Cells with MNi [per 1000 BN cells]	10.71 ± 5.25	9.18 ± 3.91	13.68 ± 6.22	**<0.001**
Total number of MNi [per 1000 BN cells]	11.9 ± 5.9	10.3 ± 4.6	14.9 ± 7.0	**<0.001**
Nuclear buds	2.62 ± 1.67	2.60 ± 1.71	2.67 ± 1.62	0.713
Nucleoplasmic bridges	1.04 ± 1.08	1.05 ± 1.02	1.00 ± 1.21	0.550
Apoptotic cells	6.06 ± 5.52	6.88 ± 5.94	4.47 ± 4.23	**<0.001**
Necrotic cells	3.85 ± 2.89	4.00 ± 2.97	3.55 ± 2.75	0.388
Nuclear division index	1.70 ± 0.23	1.75 ± 0.25	1.62 ± 0.12	**0.002**
**Body composition**	n = 100	n = 67	n = 33	
BMI [kg/m^2^]	27.3 ± 4.7	27.1 ± 4.5	27.7 ± 5.2	0.772
Waist to Hip Ratio	0.94 ± 0.13	0.98 ± 0.14	0.86 ± 0.06	**<0.001**
Lean body mass [kg]	62.2 ± 12.1	68.40 ± 9.4	50.0 ± 5.9	**<0.001**
Body cell mass[kg]	29.8 ± 6.5	33.2 ± 4.8	23.2 ± 3.5	**<0.001**
Body fat [kg]	19.3 ± 9.0	17.1 ± 7.5	23.5 ± 10.3	**0.002**
Body fat [%]	23.2 ± 8.3	19.4 ± 5.7	30.7 ± 7.6	**<0.001**
**Functional parameters**	n = 100	n = 67	n = 33	
Arm curl dominant arm 30s [repetitions]	18.5 ± 3.8	19.0 ± 4.0	17.6 ± 3.4	0.229
Timed up and go [s]	4.79 ± 0.86	4.65 ± 0.78	5.09 ± 0.96	**0.017**
6-min walk test [m]	629 ± 92	650 ± 93	585 ± 74	**<0.001**
Chair-stand 30s [repetitions]	12.5 ± 2.3	12.6 ± 2.3	12.2 ± 2.4	0.473
Handgrip dominant hand [kg]	37.7 ± 9.7	42.4 ± 7.3	27.9 ± 5.7	**<0.001**
**Blood parameters**	n = 100	n = 67	n = 33	
Erythrocytes [T/L]	4.80 ± 0.41	4.95 ± 0.37	4.47 ± 0.27	**<0.001**
Lymphocytes rel [%]	30.8 ± 8.2	30.5 ± 9.2	31.5 ± 5.6	0.157
Hemoglobin [g/dl]	14.9 ± 1.4	15.5 ± 1.2	13.7 ± 0.8	**<0.001**
Hematocrit [%]	43.0 ± 3.8	44.4 ± 3.4	40.0 ± 2.5	**<0.001**
hs-CRP [mg/L]	2.54 ± 3.98	2.43 ± 4.41	2.78 ± 2.98	0.342
FRAP [μmol/L]	1101 ± 184	1132 ± 191	1035 ± 150	**0.026**
GSH [[μmol/L]	16.60 ± 3.2	16.64 ± 3.3	16.59 ± 3.1	0.759
GSSG [μmol/L]	8.88 ± 1.59	8.83 ± 1.62	8.98 ± 1.53	0.462
GSH:GSSG ratio	1.91 ± 0.41	1.92 ± 0.41	1.89 ± 0.41	0.587
MDA [μmol/L]	2.21 ± 0.46	2.26 ± 0.49	2.11 ± 0.39	0.086
hs-Troponin [ng/L]	5.27 ± 3.13	6.00 ± 3.47	3.77 ± 1.42	**<0.001**

Data are presented as means ± standard deviation. P-values (p < 0.05) were calculated using Mann-Whitney *U* test and chi-square for measuring sex differences, significant differences are highlighted with bold numbers.

**Table 2:** Baseline characteristics based on the 50th percentile cut-off for the total number of micronuclei per 1000 BN cells in male participants.

Parameter	All	LMF	HMF	p-value
Subjects	n = 64	n = 32	n = 32	
	Mean ± SD	Mean ± SD	Mean ± SD	
**CBMN parameter**				
Cells with MNi [per 1000 BN cells]	9.2 ± 3.9	6.48 ± 1.49	11.88 ± 3.72	**<0.001**
Total number of MNi [per 1000 BN cells]	10.3 ± 4.6	7.15 ± 1.45	13.42 ± 4.49	**<0.001**
Nuclear buds	2.6 ± 1.71	2.27 ± 1.71	2.94 ± 1.67	0.077
Nucleoplasmic bridges	1.1 ± 1.02	0.98 ± 1.02	1.13 ± 1.05	0.279
Apoptotic cells	6.88 ± 5.94	6.92 ± 7.46	6.83 ± 4.02	0.862
Necrotic cells	4.00 ± 2.97	3.56 ± 2.46	4.44 ± 3.38	0.916
Nuclear division index	1.75 ± 0.25	1.74 ± 0.21	1.76 ± 0.29	0.931
**Body composition**				
BMI [kg/m^2^]	27.1 ± 4.5	25.8 ± 3.4	28.4 ± 5.1	**0.025**
Waist to Hip Ratio	0.98 ± 0.14	0.98 ± 0.17	0.98 ± 0.06	0.113
Lean body mass [kg]	68.4 ± 9.4	67.0 ± 9.0	70.0 ± 9.4	0.183
Body cell mass[kg]	33.2 ± 4.8	32.7 ± 4.6	33.6 ± 5.0	0.592
Body fat [kg]	17.1 ± 7.5	15.02 ± 5.8	19.0 ± 8.4	0.068
Body fat [%]	19.4 ± 5.6	17.9 ± 5.3	20.6 ± 5.8	0.054
**Functional parameters**				
Arm curl dominant arm 30s [repetitions]	19.0 ± 4.0	20.2 ± 4.5	17.8 ± 3.2	0.056
Timed up and go [s]	4.65 ± 0.78	4.47 ± 0.59	4.84 ± 0.95	0.129
6-min-walk test [m]	650 ± 93	680 ± 90	618 ± 90	**0.043**
30s-chair stand test [repetitions]	12.6 ± 2.3	13.4 ± 1.9	11.9 ± 2.5	**0.017**
Handgrip dominant hand [kg]	42.4 ± 7.3	42.8 ± 8.5	41.3 ± 5.8	0.726
**Blood parameters**				
Vitamin D serum level (ng/mL)	22.8 ± 5.5	22.54 ± 5.6	22.24 ± 6.24	0.220
Erythrocytes [T/L]	15.5 ± 1.2	15.5 ± 1.1	15.4 ± 1.4	0.980
Hemoglobin [g/dl]	44.4 ± 3.4	44.6 ± 3.2	44.3 ± 3.8	0.541
Hematocrit [%]	2.43 ± 4.41	1.71 ± 2.05	3.24 ± 5.99	0.548
hs-CRP [mg/L]	6.00 ± 3.47	6.00 ± 4.24	6.08 ± 2.73	**0.011**
FRAP [μmol/L]	1132 ± 191	1162 ± 186	1112 ± 204	0.277
GSH [μmol/L]	16.59 ± 3.27	16.55 ± 3.28	16.44 ± 3.34	0.727
GSSG [μmol/L]	8.83 ± 1.62	8.75 ± 1.32	8.78 ± 1.93	0.925
GSH:GSSG ratio	1.92 ± 0.42	1.92 ± 0.42	1.93 ± 0.43	0.851
MDA [μmol/L]	2.26 ± 0.49	2.21 ± 0.48	2.30 ± 0.52	0.347
hs-Troponin [ng/L]	4.95 ± 0.38	4.95 ± 0.37	4.96 ± 0.39	0.432

Data are presented as mean ± standard deviation. P-values (p < 0.05) were calculated using the Mann-Whitney *U* test, HMF group refers to the participants with greater MNi frequency than the Median of total MNi, and LMF refers to the group with less MNi frequency than the Median of total MNi.

### 3.3 Baseline correlations between MNi frequency and anthropometry, functional fitness, and blood parameters

The total number of MNi correlated significantly for the whole study population at baseline with anthropometric parameters (BMI: r = 0.311, p = 0.002, body fat (%): r = 0.433, p < 0.001, waist to hip ratio: r = −0.205, p = 0.044). There were also significant correlations with functional parameters. For instance, the arm curl test and the chair-stand test showed significant negative correlations and the 6-min walk test a positive correlation with the MNi frequency (Table 3). Also, blood parameters such as erythrocytes (T/L), hemoglobin (g/dl) and creatinine [mg/dl] correlated negatively with total MNi frequency. Separated by sex only the BMI correlated consistently with MNi (females: r = 0.369, p = 0.034; males: r = 0.265, p = 0.035). In females body fat (%) correlated positively and the chair-stand test showed a significant negative correlation with the total frequency of MNi (Table 3). No correlations between the total number of MNi and oxidative stress parameters could be observed.

**Table 3:** Correlations between MNi and health related biomarker at baseline.

Parameters	Total number of MNi All (n = 97)		Total number of MNi Females (n = 33)		Total number of MNi Males (n = 64)	
	R	p-value	R	p-value	R	p-value
**Body composition**						
BMI [kg/m^2^)	0.311**	**0.002**	0.369*	**0.034**	0.265*	**0.035**
Waist to Hip Ratio	−0.205*	**0.044**	−0.128	0.478	0.011	0.930
Lean body mass [kg]	−0.174	0.092	0.630	0.727	0.920	0.476
Body cell mass [kg]	−0.221*	**0.012**	0.206	0.251	0.129	0.319
Body fat [kg]	0.372**	**<0.001**	0.063	0.727	0.092	0.476
Body fat [%]	0.433**	**<0.001**	0.375*	**0.031**	0.186	0.148
**Functional parameters**						
Arm curl dominant arm 30s [repetitions]	−0.242*	**0.012**	−0.173	0.335	−0.223	0.079
Timed up and go [s]	−0.245*	**0.015**	0.308	0.081	0.053	0.676
6-min walk test [m]	0.262**	**0.010**	−0.256	0.151	−0.114	0.371
30s-chair stand test [repetitions]	−0.256*	**0.019**	0.347*	**0.048**	−0.159	0.210
Handgrip dominant hand [kg]	0.282**	**0.005**	−0.016	0.932	−0.040	0.751
**Blood parameters**						
Erythrocytes [T/L]	−0.264**	**0.009**	−0.088	0.625	−0.077	0.546
Hemoglobin [g/dl]	−0.345**	**0.001**	−0.266	0.135	−0.120	0.345
Hematocrit [%]	−0.309**	**0.002**	−0.174	0.334	−0.126	0.320
hs-CRP [mg/L]	0.125	0.196	0.308	0.081	0.038	0.763
FRAP [μmol/L]	−0.105	0.309	−0.010	0.956	−0.005	0.970
GSH [μmol/L]	−0.110	0.288	−0.222	0.222	−0.066	0.604
GSSG [μmol/L]	−0.137	0.182	−0.329	0.066	−0.076	0.548
GSH:GSSG ratio	0.026	0.800	0.086	0.640	0.014	0.912
MDA [μmol/L]	0.183	0.072	0.340	0.053	0.230	0.067
hs-Troponin [ng/L]	−0.196	0.054	−0.058	0.750	−0.078	0.540

**p < 0.01; *p < 0.05.

### 3.4 Influence of the intervention on vitamin D

As shown in Table 4, the vitamin D serum levels increased significantly only in the VDM group between T2 and T3 of intervention (+28 ± 14%; p < 0.001) and between T1 and the end of the study (+39 ± 14%, p < 0.001). However, the VDD group also showed a non-significant increase by +17 ± 27% in vitamin D serum levels between T1 and T3. In contrast, the control group showed no significant change in vitamin D serum levels.

**Table 4:** Development of CBMN parameters and important health biomarker after vitamin D intervention and resistance training (n = 67).

Parameter	Group	Mean ± SD			p-value	p-value			% Variation			p-value
		T1	T2	T3	Friedman	T1-T2	T2 -T3	T1-T3	%-T1-T2	%-T2-T3	%-T1-T3	ΔT1-T2 vs.ΔT2-T3
**CBMN parameters** (n = 67)												
Cells with MNi [per 1000 BN cells]	CON	10.4 ± 3.9	10.3 ± 6.5	12.8 ± 6.2	**<0.001**	0.383	0.008	0.027	−1.0 ± 66.7	24.3 ± 4.6	23.1 ± 59.0	0.065
	VDD	11.4 ± 8.2	9.02 ± 6.1	11.6 ± 4.2	**0.041**	0.110	0.039	0.313	−20.9 ± 25.6	28.6 ± 31.1	1.8 ± 48.8	**0.022**
	VDM	10.6 ± 3.9	9.0 ± 3.5	14.0 ± 6.8	**<0.001**	0.049	**0.001**	**0.001**	−15.1 ± 10.3	55.6 ± 94.3	32.1 ± 74.4	**0.003**
Total number of MNi [per 1000 BN cells]	CON	11.4 ± 4.3	11.25 ± 7.4	14.5 ± 7.2	**0.002**	0.413	**0.005**	0.025	−1.3 ± 72.1	28.9±-2.7	27.2 ± 67.4	0.051
	VDD	12.5 ± 9.2	10.0 ± 6.8	13.3 ± 5.3	**0.030**	0.217	0.047	0.179	−20.0 ± 26.1	33±-22.1	6.4±-42.4	**0.044**
	VDM	11.8 ± 4.5	10.3 ± 4.8	16.1 ± 7.8	**<0.001**	0.059	**0.001**	**<0.001**	−12.7 ± 6.7	56.3 ± 62.5	36.4 ± 73.3	**0.003**
Nuclear buds	CON	2.75 ± 1.35	3.5 ± 1.79	2.93 ± 2.36	0.102	0.054	0.143	0.919	27.3 ± 32.6	−16.3 ± 31.8	6.5 ± 74.8	0.113
	VDD	2.81 ± 2.09	3.76 ± 2.25	2.67 ± 1.93	**0.047**	0.098	0.055	0.793	33.8 ± 7.7	−29 ± 14.2	−5.0 ± 7.7	0.054
	VDM	2.46 ± 1.44	3.92 ± 2.76	2.58 ± 1.32	**0.033**	0.014	0.014	1000	59.3 ± 91.7	−34.2 ± 52.2	4.9±-8.3	**0.011**
Nucleoplasmic bridges	CON	1.02 ± 0.91	0.77 ± 0.75	0.73 ± 0.86	0.343	0.463	0.715	0.269	−24.5 ± 17.6	−5.2 ± 14.7	−28.4±-5.5	0.597
	VDD	1.31 ± 1.44	0.43 ± 0.43	1.00 ± 0.69	**0.013**	0.009	**0.004**	0.344	−67.2 ± 70.1	132.6 ± 60.5	−23.7±-52.1	**0.001**
	VDM	0.77 ± 0.92	0.88 ± 0.80	0.54 ± 0.72	0.107	0.734	0.050	0.228	14.3±-13.0	−38.6±-10.0	−29.9±-21.7	0.246
Apoptotic cells	CON	5.20 ± 4.23	6.18 ± 4.62	8.11 ± 6.37	0.063	0.154	0.071	0.048	18.8 ± 9.2	31.2 ± 37.9	56 ± 50.6	0.543
	VDD	5.40 ± 4.24	9.67 ± 8.84	7.48 ± 5.02	0.089	0.117	0.525	0.013	79.1 ± 108.5	−22.6±-43.2	38.5 ± 18.4	0.404
	VDM	7.38 ± 6.97	6.98 ± 7.17	7.98 ± 3.97	0.314	0.943	0.223	0.338	−5.4 ± 2.9	14.3±-44.6	8.1±-43.0	0.368
Necrotic cells	CON	3.70 ± 2.70	4.16 ± 2.25	4.89 ± 3.98	0.825	0.464	0.564	0.286	12.4 ± 16.7	17.5 ± 76.9	32.2 ± 47.4	0.972
	VDD	3.64 ± 2.94	5.26 ± 4.51	5.71 ± 4.05	0.088	0.172	0.588	0.01	44.5 ± 53.4	8.6 ± 10.2	56.9 ± 37.8	0.626
	VDM	5.00 ± 3.60	3.71 ± 2.83	3.19 ± 2.37	0.153	0.143	0.519	0.048	−25.8±-21.4	−14 ± 16.3	−36.2 ± 34.2	0.391
Nuclear division index	CON	1.70 ± 0.36	1.66 ± 0.17	1.77 ± 0.27	0.861	0.627	0.101	0.445	−2.4 ± 52.8	6.6 ± 58.8	4.1 ± 25.0	0.131
	VDD	1.72 ± 0.10	1.70 ± 0.38	1.78 ± 0.34	0.827	0.848	0.339	0.375	−1.2 ± 280.0	4.7 ± 10.5	3.5 ± 240	0.434
	VDM	1.69 ± 0.20	1.64 ± 0.14	1.68 ± 0.19	0.325	0.317	0.361	0.458	−3.0 ± 30.0	2.4 ± 35.7	−0.6 ± 5.0	0.209
**Changes in Blood parameters** (n = 67)												
Vitamin D serum level [ng/mL]	CON	20.8 ± 4.8	21.2 ± 5.1	23.6 ± 8.3	0.970	0.685	0.123	0.088	1.9 ± 6.3	11.3 ± 62.7	13.5 ± 72.9	0.168
	VDD	23.9 ± 6.0	24.4 ± 6.6	28.0 ± 7.6	**0.010**	0.444	0.025	0.023	2.1 ± 10.0	14.8 ± 15.2	17.2 ± 26.7	0.181
	VDM	23.7 ± 6.6	25.8 ± 4.6	32.9 ± 7.6	**<0.001**	0.008	**<0.001**	**<0.001**	8.9 ± 30.3	27.5 ± 65.2	38.8 ± 15.2	**0.013**
hs-CRP [mg/L]	CON	2.51 ± 3.04	2.33 ± 3.75	3.01 ± 6.28	0.190	0.531	0.374	0.156	−7.2 ± 23.4	29.2 ± 67.5	19.9 ± 106.6	0.862
	VDD	3.46 ± 6.73	2.18 ± 1.81	2.12 ± 1.59	0.604	0.777	0.736	0.851	−37.0 ± 73.1	−2.8 ± 12.2	−38.7 ± 76.4	0.835
	VDM	1.77 ± 1.74	1.79 ± 2.24	1.49 ± 1.46	0.059	0.985	0.242	0.116	1.1 ± 28.7	−16.8±-34.8	−15.8 ± 16.1	0.664
FRAP [μmol/L]	CON	1143 ± 221	1044 ± 137	941 ± 175	**<0.001**	0.025	**0.003**	**<0.001**	−8.7 ± 38	−9.9 ± 27.7	−17.7 ± 20.8	0.958
	VDD	1144 ± 191	1100 ± 150	986 ± 217	**<0.001**	0.235	**0.004**	**<0.001**	−3.8 ± 21.5	−10.4 ± 44.7	−13.8 ± 13.6	0.136
	VDM	1123 ± 197	1117 ± 210	965 ± 210	**<0.001**	0.455	**<0.001**	**<0.001**	−0.5 ± 6.6	−13.6 ± 0.0	−14.1 ± 6.06	**0.003**
GSH [μmol/L]	CON	16.61 ± 2.33	15.65 ± 1.60	14.70 ± 1.82	0.013	0.024	0.110	**0.002**	−6.0 ± 31.3	−5.8 ± 13.8	−11.4 ± 21.9	0.986
	VDD	16.32 ± 3.09	15.63 ± 2.05	16.00 ± 3.55	0.175	0.372	0.896	0.478	−4.3 ± 33.7	2.6 ± 73.2	−1.8 ± 14.9	0.777
	VDM	16.13 ± 0.44	14.90 ± 2.49	14.92 ± 2.75	0.063	0.040	0.821	0.050	−7.5 ± 465.9	−0.1 ± 10.4	−7.5 ± 52.5	0.366
**Changes in Blood parameters** (n = 67)												
GSSG [μmol/L]	CON	9.37 ± 1.52	8.61 ± 1.03	8.46 ± 1.51	0.190	0.012	0.543	0.009	−8.1 ± 32.2	−1.7 ± 46.6	−9.7 ± 0.7	0.297
	VDD	8.06 ± 1.79	8.03 ± 1.43	8.23 ± 1.41	0.604	0.962	0.306	0.653	−0.4 ± 20.1	2.5 ± 1.4	2.1 ± 21.2	0.542
	VDM	8.82 ± 1.58	7.83 ± 1.24	8.01 ± 1.57	**0.004**	**0.004**	0.414	0.156	−11.2 ± 21.5	2.3 ± 26.6	−9.2 ± 0.6	**0.008**
GSH/GSSG ratio	CON	1.80 ± 0.32	1.83 ± 0.26	1.80 ± 0.43	0.937	0.903	0.737	0.917	1.7 ± 18.8	−1.6 ± 65.4	0 ± 34.4	0.664
	VDD	2.27 ± 0.45	1.95 ± 1.07	2.38 ± 0.78	0.326	0.286	0.408	0.102	−14.1 ± 137.8	22.1 ± 27.1	4.8 ± 73.3	1.000
	VDM	1.87 ± 0.43	1.95 ± 0.40	1.89 ± 0.29	0.419	0.537	0.445	0.974	4.3 ± 7.0	−3.1 ± 27.5	1.1 ± 32.6	0.390
MDA [μmol/L]	CON	2.11 ± 0.58	1.75 ± 0.99	2.45 ± 0.79	0.190	0.091	0.033	0.274	−17.1 ± 70.7	40 ± 20.2.0	16.1 ± 36.2	**0.009**
	VDD	2.25 ± 0.48	2.04 ± 1.02	2.29 ± 0.78	0.604	0.353	0.554	0.872	−9.3 ± 112.5	12.3 ± 23.5	1.8 ± 62.5	0.084
	VDM	2.25 ± 0.43	2.18 ± 1.24	2.28 ± 0.77	0.059	0.532	0.692	0.857	−3.1 ± 188.4	4.6 ± 37.9	1.3 ± 79.1	0.378
hs-Troponin [ng/L]	CON	5.74 ± 5.07	5.47 ± 4.59	4.96 ± 4.33	0.068	0.355	0.229	0.014	−4.7 ± 9.5	−9.3 ± 5.7	−13.6 ± 14.6	0.745
	VDD	5.14 ± 2.11	5.30 ± 2.22	5.04 ± 3.10	**0.004**	0.590	0.067	0.067	3.1 ± 5.2	−4.9 ± 39.6	−1.9 ± 46.9	0.211
	VDM	5.42 ± 2.48	5.53 ± 3.92	5.23 ± 3.32	0.400	0.628	0.919	0.330	2.0 ± 58.1	−5.4 ± 15.3	−3.5 ± 33.9	0.932
**Changes in body composition after vitamin D intervention and resistance training** (n = 67)												
BMI [kg/m^2^]	CON	26.2 ± 4.9	26.1 ± 4.8	26.1 ± 5.1	0.708	0.424	0.719	0.387	−0.4 ± 2.0	0.0 ± 6.3	−0.4 ± 4.1	0.987
	VDD	28.3 ± 4.4	28.2 ± 4.5	28.0 ± 4.9	0.163	0.360	0.548	0.084	−0.4 ± 2.3	−0.7 ± 8.9	−1.1 ± 11.4	0.322
	VDM	26.8 ± 4.3	26.7 ± 4.4	26.6 ± 4.6	0.508	0.819	0.399	0.264	−0.4 ± 2.3	−0.4 ± 4.5	−0.7 ± 7.0	0.841
Waist to Hip Ratio	CON	0.92 ± 0.08	0.92 ± 0.08	0.92 ± 0.09	0.618	0.325	0.471	0.486	0.0 ± 0.0	0.0 ± 12.5	0.0 ± 12.5	0.833
	VDD	0.98 ± 0.22	0.94 ± 0.09	0.94 ± 0.09	0.772	0.848	0.581	0.923	−4.1 ± 59.1	0.0 ± 0.0	−4.1 ± 59.1	0.958
	VDM	0.93 ± 0.07	0.93 ± 0.08	0.92 ± 0.07	0.679	0.695	0.367	0.468	0.0 ± 14.3	−1.1 ± 12.5	−1.1 ± 0.0	0.493
Lean body mass [kg]	CON	59.9 ± 11.8	60.1 ± 12.0	61.0 ± 14.3	0.306	0.448	0.104	0.076	0.3 ± 1.7	1.5 ± 19.2	1.8 ± 21.2	0.744
	VDD	63.3 ± 12.3	63.9 ± 12.5	64.5 ± 12.9	0.113	0.141	0.082	0.033	0.9 ± 1.6	0.9 ± 3.2	1.9 ± 4.9	0.130
	VDM	62.9 ± 12.5	62.8 ± 12.8	64.0 ± 12.8	0.134	0.770	0.019	0.025	−0.2 ± 2.4	1.9 ± 0	1.7 ± 2.4	0.104
Body cell mass [kg]	CON	28.4 ± 6.5	29.2 ± 6.7	29.6 ± 6.7	0.232	0.079	0.417	0.044	2.8 ± 3.1	1.4 ± 0	4.2 ± 3.1	0.794
	VDD	30.8 ± 6.8	31.0 ± 6.7	31.6 ± 7.3	0.354	0.303	0.158	0.082	0.6 ± 1.5	1.9 ± 9	2.6 ± 7.4	0.350
	VDM	30.1 ± 6.6	30.0 ± 6.7	30.6 ± 6.3	0.047	0.657	0.063	0.040	−0.3 ± 1.5	2.0 ± 6.0	1.7 ± 4.5	0.117
Body fat [kg]	CON	18.7 ± 86	18.2 ± 9.2	16..9 ± 9.1	0.199	0.164	0.111	0.042	−2.7 ± 89.3	−6.7 ± 11.5	−8.1 ± 18.0	0.983
	VDD	20.4 ± 8.8	19.4 ± 8.6	18.3 ± 8.2	**<0.001**	**0.002**	**0.002**	**<0.001**	−4.9 ± 2.3	−5.7 ± 4.7	−10.3 ± 6.8	0.728
	VDM	18.8 ± 8.2	18.6 ± 7.9	17.0 ± 8.5	**0.001**	0.831	**<0.001**	**<0.001**	−1.1 ± 3.7	−8.6 ± 7.6	−9.6 ± 3.7	**0.043**
Body fat [%]	CON	23.3 ± 7.0	22.7 ± 8.2	21.7 ± 8.1	0.244	0.167	0.098	0.036	−2.6 ± 17.1	−4.4 ± 1.2	−6.9 ± 15.7	0.879
	VDD	24.1 ± 8.5	23.0 ± 8.7	21.8 ± 8.3	**<0.001**	**0.005**	**0.002**	**<0.001**	−4.6 ± 2.4	−5.2 ± 4.6	−9.5 ± 2.4	0.351
	VDM	22.6 ± 7.4	22.5 ± 7.7	20.5 ± 7.8	**0.001**	0.750	**<0.001**	**<0.001**	−0.4 ± 4.1	−8.9 ± 1.3	−9.3 ± 5.4	**0.019**

Data are presented as mean ± standard deviation. Friedman test was used to calculate differences over three time points with a P-value (p < 0.05). Differences between two time points were calculated using the Mann-Whitney *U* test with a P-value (p < 0.005) (Bonferroni corrected). Wilcoxon test was used to calculate the differences (Δ) between phase 1 (T2-T1) and differences (Δ) phase 2 (T3-T2) (p < 0.05). Bold numbers indicate significant differences. CON refers to control group, VDD refers to Vitamin D daily group (800 IU daily), VDM refers to Vitamin D monthly group (50.000 IU monthly).

### 3.5 Intervention effects on chromosomal stability

Data of 67 participants were analyzed that completed all three main time points, T1, T2 and T3, as can be seen in Table 4. At baseline, there were no statistical differences between the intervention groups (CON, VDD, VDM) when it comes to parameters related to the CBMN assay. Also, the anthropometric parameters did not differ significantly between the three intervention groups (CON, VDD, VDM; Table 4). To assess the effect of vitamin D intervention and physical activity on chromosomal stability, parameters of the CBMN assay were analyzed. After 4 weeks of intervention, the total number of micronuclei was reduced in all the three groups, however, the strongest reduction could be observed in the group receiving 800 IU vitamin D daily (−21 ± 14%). Interestingly, an increase in total numbers of MNi frequencies was observed in all study groups at T3. After 17 weeks of intervening with vitamin D supplementation combined with 10 weeks of resistance training, we observed a statistically significant increase in the MNi frequencies in the VDM group and the CON group compared to baseline. At T3, the VDD group showed the lowest increase in MNi frequency (+7 ± 42%; p = 0.001). The biggest increase in MNi was found in the VDM group +36 ± 73%, followed by the CON group +27 ± 67%. When looking at the differences between phase 1 (T2-T1) and phase 2 (T3-T2), MNi in the VDD and VDM group differed significantly (Table 4). Additionally, the number of nuclear buds was reduced by −5 ± 8% in the VDD group at T3, whereas it was non-significantly increased in the CON group by +7 ± 75% as well as in the VDM group by +5 ± 8% after 17 weeks. Significant phase difference related to NBDs could be measured in the VDM group (Table 4). The number of nucleoplasmic bridges was reduced significantly only in the VDD group between T1 and T3, as shown in Table 4. No significant differences were observed between intervention groups regarding the remaining chromosomal stability parameters including, apoptotic cell or necrotic cells and the overall nuclear division index.

### 3.6 Effect of the intervention on oxidative stress parameters: FRAP, GSH/GSSG and MDA

GSH activity was significantly reduced in the CON group between T1 and T3 over the whole intervention period (Table 4). The VDM group showed decreasing non-significant tendencies in the mean GSH levels between T1 and T3. Analysis of GSSG revealed significant differences in the VDM group between the two study phases (T2–T1 vs. T3-T2). GSSG showed significant differences in the VDM group between the supplementation phase (T1-T2) with a reduction of −11.2 ± 21.5% of GSSG activity levels. Similar but not significant tendencies for GSSG could be observed in the CON group. In the VDM group no significant changes could be observed regarding GSSG activity. GSH:GSSG ratio did not change significantly in the three groups (CON, VDD, VDM). FRAP antioxidant capacity significantly decreased in all three intervention groups between T1 and T3 with the largest decrease in the CON group (−17.7 ± 20.8%), followed by VDM and VDD respectively (Table 4). Especially between T2 and T3, the strongest decrease in FRAP levels could be observed in the VDM group (−13.6 ± 0%). MDA levels showed no significant changes over time; but the analyses of the phase differences (Δ) showed that in Δ phase 1 (T2-T1), the mean MDA difference was negative in all groups (CON, VDD, VDM). However, in Δ phase 2 (T3-T2) the mean MDA differences were positive in all groups and statistical analyses revealed that the comparison of Δ phase 1 with Δ phase 2 showed significant differences in the CON group, but not in VDD and VDM (Table 4).

### 3.7 Changes of functional fitness parameters after the intervention

The very detailed results regarding the strength training intervention is published by Aschauer et al. [18]. Performance parameters, such as the 30-s chair stand test as well as the arm curl test increased over time in all groups after the resistance exercise intervention, especially in the 2nd phase of the study between T2 and T3. Parameters, such as the 6-min walk test, timed up and go improved over time between T1 and T3 (Table 2 Supplementary). However, in this study none of the parameters related to physical performance were influenced by the different vitamin D supplementation regimen as can be seen in Table 2 Supplementary.

## 4 Discussion

The main goal of the presented analyses of the Vitamin D NutriAging study was to investigate the effect of a 17-week vitamin D supplementation regimen combined with 10 weeks of strength training, on parameters regarding chromosomal damage and instability using CBMN assay in a cohort of older community-dwelling women and men in Vienna [18]. The sex distribution in this analysis was 67% men and 33% women. The BMI of the participants was slightly elevated with an average of 27 kg/m^2^ (Table 1) when compared to younger people, however, in older adults, a BMI categorized as “overweight” might rather be advantageous in terms of mortality risk [58,59]. The MNi frequency in PBMCs correlated positively with BMI (Table 3) which is in accordance with literature [60,61]. Further, MNi frequency was higher in females than in males, as already shown previously (Table 1) [[61], [62], [63]]. Furthermore, we observed consistent significant correlations between MNi frequency and physical performance parameters in our study cohort (Table 3). We were able to recruit more men than women in this study and interestingly, men with higher MNi frequencies showed also worse performance in certain functional parameters such as the 6-min walk test and the 30-s chair stand test compared to those men with lower MNi frequencies indicating that a greater chromosomal instability might be related to poorer functional fitness. However, this effect was not observed in the female participants, except for the number of nuclear buds. Females displayed 36.4% less nuclear buds in the group with higher MNi frequencies than those with less MNi numbers (50th percentile cut-off). Nuclear buds are assumed to have different mechanistic origin compared with micronuclei. Cell culture experiments indicate that nuclear buds might be influenced by folate status [23]. Various studies and metanalyses investigated the effect of different doses of vitamin D supplementation in older people [31,37,34,64]. However, literature on vitamin D status, vitamin D supplementation and chromosomal damage is scarce. Only one study found associations of serum vitamin D levels using CBMN endpoint markers indicating, that insufficient maternal levels of 25(OH)D is associated with elevated levels of MNi frequency in cord blood [65]. Another cross-sectional study reported that serum vitamin D levels were not associated with chromosomal damage [66]. However, since studies and metanalyses show inconclusive results regarding the effects of vitamin D supplementation on age-related outcomes [[31], [32], [33], [34], [35], [36]] and vitamin D supplementation has not been investigated with respect to chromosomal damage parameters in older vitamin D deficient adults before, we divided participants into different groups: VDM, VDD and CON. The VDD study group received the recommended vitamin D supplementation of 800 IU daily according to the D-A-CH reference values for German-speaking countries [67,68]. The VDM group received vitamin D supplementation of 50.000 IU once per month as a single high dose. to investigate whether moderately elevated vitamin D levels might ameliorate chromosomal instability in vitamin D deficient older adults. Additionally, all intervention groups including the control group consumed 400 mg calcium daily over the whole intervention period. Several intervention studies and metanalyses suggest that calcium supplementation might decrease the risk for falls and consequently bone fractures in older individuals [[39], [40], [41]]. Further, the combination of calcium together with vitamin D supplementation might prevent the onset of osteoporosis in postmenopausal women and is necessary for the maintanence of a healthy muscle physiology [[39], [40], [41]]. Regarding physical exercise, studies have shown that moderate exercise can reduce MNi frequency [69], whereas intense exercise might even increase the occurrence of MNi in healthy individuals [70]. To date, there are no published studies investigating the effect of exercise and vitamin D supplementation on chromosomal stability in PBMCs of older adults. Franzke et al., reported that aerobic fitness might function as a protective factor against chromosomal instabilities in older institutionalized people [9]. However, we found that strength training significantly increased the total number of MNi in all intervention groups, with the highest rise in MNi in the VDM group. Also, in the VDD group a small increase in MNi frequency could be observed, but this was not significant (Table 4). When comparing the VDM regimen vs. the VDD regimen, the results indicate that a moderate daily intake of 800 IU per day of vitamin D might ameliorate adverse effects on chromosomal damage triggered by strength training in older individuals. Vitamin D serum levels increased the most (and only significant) in the VDM group followed by the VDD group and the control group after 17 weeks of intervention indicating a seasonal influence on serum Vitamin D levels. This could also partly be explained by the increasing exposure of the participants to UV radiation between mid-February and mid-July 2019. A positive association of UV solar exposure and elevated chromosomal DNA damage levels in adults have been reported previously [66,71]. Hence, the exposure to sunlight over the study period might also have contributed to increased chromosomal damage at the end of the intervention. As already mentioned, the effect of demanding resistance training on MNi frequency should also be considered as a major factor. In accordance with the exclusion criteria, the participants were not used to perform strength training before entering the study. As reported earlier [18], 12% of the participants with minor medical issues dropped out in the second phase of the study reporting worsened pain or other symptoms when the resistance exercise period started. Interestingly, the mean number of MNi was (not significantly) lowered in all groups in the first 4 weeks without exercise. However, the increase in MNi frequency could be observed in all groups especially after finishing the strength training intervention at T3. Resistance exercise is known to influence oxidative stress parameters in blood and skeletal muscle tissue [72]. Reactive oxygen species are signaling molecules that are being formed especially after a high oxygen demanding training as a result of adaption to the training. Nonetheless, in people who are not accustomed properly to resistance workout, an intensively performed training over a longer time period can result in chronic oxidative stress causing chromosomal instability [73]. On the other hand, exercise can also be protective against chromosomal instability by upregulating DNA repair mechanisms as well as the activity of antioxidant enzymes that are induced by regular training sessions [74]. Interestingly, various oxidative stress markers adversely changed over the course of the intervention time, especially between T2 and T3. For example, GSH is one of the most important endogenous cellular antioxidants and is known to exert protective cellular effects [29]. GSH was reduced in all study groups but at the end of the intervention, the VDD group showed the smallest reduction tendencies in GSH levels of only 1.8% compared to the CON (−11%) and VDM group (−7%). Further, the VDD group was the only intervention group that showed increasing tendencies of the mean GSH activities in the second phase of the intervention (T2-T3) when the vitamin D supplementation and the sport intervention was combined (Table 4). This would indicate a protective effect for recommended daily vitamin D supplementations in vitamin D deficient older adults new to regular resistance exercise. Although the GSH:GSSG ratio did not alter significantly, some interesting tendencies could be observed. For example, VDD was the only group that displayed an increase in the GSH:GSSG ratio when the strength training intervention started, particularly between T2 and T3 (+22.1 ± 27.1%) and at the end of the whole intervention period (+4.8 ± 7.3%) indicating improved oxidative stress protection [75]. Interestingly, the antioxidant capacity determined by measuring FRAP was reduced in all study groups, but the least reduction could be observed in the VDD and VDM groups compared to the control group. Further, FRAP antioxidant capacity decreased significantly in all intervention groups especially between T2 and T3 as well as over the whole study period. These results indicate that the intensive training protocol in this formerly untrained age-group might have contributed to the detrimental effects of elevated oxidative stress on chromosomal stability parameters. However, at T3 the least reduction of FRAP values could be measured in the VDD and the VDM group (+13.8% and +14.1% respectively), while the biggest increase in the CON group with 17.1% indicating a protective effect of vitamin D supplementation to a certain level [75]. Additionally, although plasma MDA levels, which are well-known oxidative stress marker [27], were not significantly altered by the intervention, the results revealed a rise of 16% in the mean of MDA levels in the CON group compared to the vitamin D supplementation groups (VDD: 1.8% and VDM: 1.3%). Only in the CON group the differences of mean MDA levels between phase 1 and phase 2 were significant (Table 4) This indicates that the vitamin D intake in VDD and VDM diminished adverse alterations of MDA between the supplementation period only vs. the training-and supplementation period. Thus, vitamin D supplementation could not prevent but it could ameliorate some of the adverse effects of resistance exercise that was experienced by the majority of the participants as an intense training throughout the second phase of the study. This might have been detrimental regarding the total number of MNi indicating elevated chromosomal instability in older, otherwise healthy subjects, that might be due to the fact that these participants did not experience age-related strength training before entering this study. Only very few studies focused on frequencies of nucleoplasmic bridges (NPBs) and further chromosomal stability parameters of the CBMN assay. NPBs are considered as direct evidence for chromosomal instability stemming from for misrepair of DNA strand breaks. They did not change significantly in the control group and the VDM group. However, in the VDD group there was a non-significant decrease in the mean value of NPBs by 67% after 4 weeks of intervention with 800 IU vitamin D daily solely. After 17 weeks intervention with vitamin D and resistance training, a significant reduction in NPB frequency by 24% could be detected in the VDD group compared to baseline. This decrease of NPBs indicate that the supplementation regimen in the VDD of 800 IU per day might result in better removal of excessed amplified DNA appearing as NPBs under the microscope [23]. In contrast to this, there were no significant effects on the number of NPBs in the CON and VDM group. After 17 weeks of vitamin D supplementation alone or in combination with resistance training, no difference in any other parameter of the CBMN assay or inflammatory parameters such as hs-CRP levels could be detected (Table 4). Also, a recently published vitamin D human intervention study showed no dose-dependent effects of vitamin D supplementation, combined with omega-3 fatty acids and strength training on blood pressure, physical performance, and infections [64]. Further, dose-dependent vitamin D supplementation did not alter bone mineral density in older individuals [77]. There are several factors that may account for the lack of a greater effect of the vitamin D intervention in our study. Despite the fact that our subjects displayed either vitamin D deficient or insufficient serum levels, they were relatively healthy and were free of acute diseases. Our study started in late winter and finished in mid-July, thus; we cannot exclude entirely dermal endogenous vitamin D syntheses, which has occurred during the intervention period in addition to the supplementation regimen. Although our results indicate certain adverse effects on parameters regarding chromosomal stability and oxidative stress on a cellular level, we could also observe improved physical function in the handgrip test, the chair stand test or the arm curl test (Table 2 Supplementary) indicating more strength in the elderly study subjects. More strength in older people is associated with increased mobility and an improved quality of life [78,79]. A longer adjustment period and a slower progression of the strength training might be needed in this age group to reach the optimal cellular adaptation to regular strength training in older, formerly untrained people.

## 5 Conclusion

Our analyses of chromosomal damage in PBMCs in a cohort of 100 community-dwelling older adults add important insights to the field of DNA stability, oxidative stress and mutagenesis. To our knowledge, we were the first who investigated the effect of different vitamin D regimens in older individuals during an ongoing strength training period of 10 weeks while receiving vitamin D supplementation at the recommended level of 800 IU per day vs. a single dose of 50.000 IU per month. Although we did not detect major effects of vitamin D supplementation on some parameters of chromosomal instability after the intervention, we observed some interesting tendencies of major chromosomal instability parameters. For instance, the number of NPBs was significantly reduced by 24% only in the vitamin D intervention group that received the recommended 800 IU daily. Moreover, the increase in MNi frequency, that might have been caused by the intensity of the strength training between T2 and T3, was the smallest in the VDD group. Interestingly, either the lack of vitamin D supplementation in the control group as well as vitamin D supplementation well above the recommended levels of 800 IU in the VDM group might have further increased chromosomal damage. Our results suggest that oxidative stress might have played a role in the detrimental progress on chromosomal stability parameters since the protective effect of GSH was reduced in all study groups at the end of the intervention, but the least reduction occurred also in the VDD group. Interestingly, the GSH:GSSG ratio increased only in the VDD group during the sports intervention. FRAP antioxidant capacity was reduced in all study groups, but the least reduction could be observed in the VDD and VDM groups at the end of the study. These results indicate that a supplementation with the recommended dose of 800 IU vitamin D per day might be more advantageous when it comes to chromosomal stability parameters such as MNi frequency and NPBs in older, formerly untrained participants undergoing demanding resistance exercise for 10 weeks. However, these effects might be more pronounced in old participants with even lower levels of vitamin D and should be considered in future studies.

## Funding

This work was supported by the 10.13039/501100003065University of Vienna, by funding the Research Platform Active Ageing, and the EU-program Interreg SK-AT (NutriAging). This article is supported by the Open Access Publishing Fund of the 10.13039/501100003065University of Vienna.

## Declaration of competing interest

The authors declare that there is no conflict of interest.
