# Supplementary material for: The influence of vitamin D supplementation and strength training on health biomarkers and chromosomal damage in community-dwelling older adults
Source: Redox Biol. 2023 Feb 21;61:102640. doi: 10.1016/j.redox.2023.102640 (PMC9986641; doi:10.1016/j.redox.2023.102640)
Supplement: Multimedia component 1 [file mmc1.docx]

| **Table 1 Supplementary**: Baseline characteristics based on the 50^th^. percentile cut-off for the total number of micronuclei per 1000 BN cells in female participants | | | | | | | | |  |
| --- | --- | --- | --- | --- | --- | --- | --- | --- | --- |
|  | | | | | | | | |  |
| Parameter |  | All |  | LMF |  | HMF |  |  | |
| Subjects |  | n = 33 |  | n = 16 |  | n = 17 |  |  | |
|  |  | Mean ± SD |  | Mean ± SD |  | Mean ± SD |  | p-value | |
| **CBMN parameter** |  |  |  |  |  |  |  |  | |
| Cells with MNi [per 1000 BN cells] |  | 13.7±6.2 |  | 15.24±7.42 |  | 12.03±4.28 |  | 0.154 | |
| Total number of MNi [per 1000 BN cells] |  | 14.9±7.0 |  | 16.56±8.36 |  | 13.13±4.94 |  | 0.182 | |
| Nuclear buds |  | 2.67±1.62 |  | 3.24±1.68 |  | 2.06±1.36 |  | **0.025** | |
| Nucleoplasmic bridges |  | 1.00±1.21 |  | 1.18±1.38 |  | 0.81±1.00 |  | 0.407 | |
| Apoptotic cells |  | 4.47±4.23 |  | 4.24±4.42 |  | 4.72±4.15 |  | 0.857 | |
| Necrotic cells |  | 3.55±2.75 |  | 3.79±2.93 |  | 3.28±2.62 |  | 0.560 | |
| Nuclear division index |  | 1.75±0.26 |  | 1.87±1.60 |  | 1.64±0.14 |  | 0.428 | |
| **Body composition** |  |  |  |  |  |  |  |  | |
| BMI [kg/m²] |  | 27.7±5.2 |  | 28.0±4.9 |  | 27.5±5.6 |  | 0.692 | |
| Waist to Hip Ratio |  | 0.86±0.06 |  | 0.87±0.06 |  | 0.86±0.06 |  | 0.64 | |
| Fat free mass BCM plus ECM [kg] |  | 50.0±5.9 |  | 49.2±4.3 |  | 50.7±7.4 |  | 0.692 | |
| Muscle mass[kg] |  | 23.2±3.5 |  | 23.1±3.5 |  | 23.3±3.7 |  | 0.679 | |
| Body fat [kg] |  | 23.5±10.3 |  | 23.1±7.7 |  | 23.9±12.7 |  | 0.759 | |
| Body fat [%] |  | 30.7±7.6 |  | 31.2±6.5 |  | 30.1±8.8 |  | 0.787 | |
| **Functional parameters** |  |  |  |  |  |  |  |  | |
| Arm curl dominant arm 30s [repetitions] |  | 17.6±3.4 |  | 17.1±3.7 |  | 18.0±3.0 |  | 0.48 | |
| Timed up and go [s] |  | 5.09±0.96 |  | 5.15±1.15 |  | 5.03±0.75 |  | 0.829 | |
| 6-min walk test [m] |  | 585±74 |  | 584±81 |  | 586 ±68 |  | 0.666 | |
| Chair-stand 30s [repetitions] |  | 12.21±2.41 |  | 12.41±2.72 |  | 12.00±2.10 |  | 0.689 | |
| Handgrip dominant arm [kg] |  | 27.9±5.7 |  | 27.9±5.4 |  | 27.8±6.3 |  | 0.885 | |
| **Blood parameters** |  |  |  |  |  |  |  |  | |
| Vitamin D serum level [ng/mL] |  | 23.67.±4.82 |  | 22.10±1.60 |  | 25.38±1.64 |  | 0.780 | |
| Erythrocytes [T/L] |  | 4.47±0.27 |  | 4.43±0.22 |  | 4.53±0.31 |  | 0.564 | |
| Hemoglobin [g/dL] |  | 13.7±0.8 |  | 13.6±0.6 |  | 13.8±1.0 |  | 0.427 | |
| Hematocrit [%] |  | 40.0±2.5 |  | 39.6±2.1 |  | 40.5±2.9 |  | 0.57 | |
| hs-CRP [mg/L] |  | 2.78±2.98 |  | 2.49±2.02 |  | 3.09±3.8 |  | 0.732 | |
| FRAP [µmol/L] |  | 1035±150 |  | 1025±153 |  | 1046±151 |  | 0.610 | |
| GSH [µmol/L] |  | 16.64±3.11 |  | 16.51±3.34 |  | 16.78±2.93 |  | 0.806 | |
| GSSG [µmol/L] |  | 8.98±1.53 |  | 8.55±1.56 |  | 9.46±1.39 |  | 0.069 | |
| GSH:GSSG ratio |  | 1.89±0.41 |  | 1.96±0.40 |  | 1.81±0.43 |  | 0.206 | |
| MDA [µmol/L] |  | 2.11±0.39 |  | 2.04±0.44 |  | 2.18±0.33 |  | 0.331 | |
| hs-Troponin [ng/L] |  | 3.77±1.42 |  | 3.44±1.38 |  | 4.14±21.41 |  | 0.160 | |
| Data are presented as mean ± standard deviation. P-values (p < 0.05) were calculated using the Mann-Whitney U test, HMF group refers to the participants with greater MNi frequency than the Median of total MNi, and LMF refers to the group with less MNi frequency than the Median of total MNi. | | | | | | | | |  |
|  | | | | | | | | |  |

**Supplementary:**

| **Table 2 Supplementary** |  |  |  |  |  |  |  |  |  |  |  |  |  |  |  |  |
| --- | --- | --- | --- | --- | --- | --- | --- | --- | --- | --- | --- | --- | --- | --- | --- | --- |
| **Changes in body composition after vitamin D intervention and resistance training** (n=67) | | | | | | | | | | | | | | | | |
| Parameter | Group |  | Mean ± SD | p-value |  | p-value |  | % Variation |  | p-value |  |  |  |  |  |  |
|  |  |  | T1 | T2 | T3 | Friedman |  | T1-T2 | T2 -T3 | T1-T3 |  | %-T1-T2 | %-T2-T3 | %-T1-T3 |  | ΔT1-T2 vs.ΔT2-T3 |
| Arm curl dominant | CON |  | 18.0±3.1 | 18.9±2.7 | 22.4±3.5 | **<0.001** |  | 0.027 | **<0.001** | **<0.001** |  | 5.0±12.9 | 18.5±29.6 | 24.4±12.9 |  | **0.006** |
| arm 30s [repetitions] | VDD |  | 19.5±5.0 | 21.1±4.9 | 23.8±5.3 | **<0.001** |  | **0.001** | **<0.001** | **<0.001** |  | 8.2±2.0 | 12.8±8.2 | 22.1±6.0 |  | 0.317 |
|  | VDM |  | 18.2±3.6 | 19.6±3.1 | 22.5±3.3 | **<0.001** |  | 0.067 | **<0.001** | **<0.001** |  | 7.7±13.9 | 14.8±6.5 | 23.6±-8.3 |  | **0.049** |
| Timed up and go [s] | CON |  | 4.78±0.70 | 4.74±0.72 | 4.67±0.82 | 0.165 |  | 0.517 | 0.198 | 0.182 |  | -0.8±2.9 | -1.5±13.9 | -2.3±17.1 |  | 0.884 |
|  | VDD |  | 4.84±0.83 | 4.63±0.85 | 4.71±0.90 | 0.116 |  | 0.018 | 0.296 | 0.225 |  | -4.3±2.4 | 1.7±5.9 | -2.7±8.4 |  | 0.093 |
|  | VDM |  | 4.74±0.90 | 4.70±0.94 | 4.60±0.86 | 0.067 |  | 0.185 | 0.112 | 0.071 |  | -0.8±4.4 | -2.1±8.5 | -3.0±4.4 |  | 0.530 |
| 6-min walk test [m] | CON |  | 620±89 | 636±89 | 636±93 | 0.104 |  | 0.041 | 0.923 | 0.058 |  | 2.6±0.0 | 0.0±4.5 | 2.6±4.5 |  | 0.088 |
|  | VDD |  | 631±96 | 637±92 | 642±107 | 0.764 |  | 0.485 | 0.889 | 0.151 |  | 1.0±4.2 | 0.8±16.3 | 1.7±11.5 |  | 0.108 |
|  | VDM |  | 651±93 | 657±93 | 657±96 | 0.187 |  | 0.126 | 0.875 | 0.308 |  | 0.9±0.0 | 0.0±3.2 | 0.9±3.2 |  | 0..361 |
| 30s-chair stand test | CON |  | 12.6±2.19 | 13.7±2.78 | 15.6±3.43 | **<0.001** |  | **0.004** | **<0.001** | **<0.001** |  | 8.7±26.9 | 13.9±23.4 | 23.8±56.6 |  | 0.242 |
| [repetitions] | VDD |  | 13.2±2.39 | 14.4±2.72 | 15.9±3.05 | **<0.001** |  | 0.007 | **0.001** | **<0.001** |  | 9.1±13.8 | 10.4±12.1 | 20.5±27.6 |  | 0.598 |
|  | VDM |  | 12.2±2.27 | 13.3±2.16 | 15.2±2.47 | **<0.001** |  | **<0.001** | **<0.001** | **<0.001** |  | 9.0±4.8 | 14.3±14.4 | 24.6±8.8 |  | 0.271 |
| Handgrip dominant | CON |  | 37.2±10.9 | 36.3±10.2 | 33.3±10.7 | **<0.001** |  | 0.061 | **<0.001** | **<0.001** |  | -2.4±6.4 | -8.3±4.9 | -10.5±1.8 |  | **0.006** |
| hand [kg] | VDD |  | 37.4±9.8 | 35.8±10.6 | 33.4±10.0 | **<0.001** |  | 0.007 | **<0.001** | **<0.001** |  | -4.3±8.2 | -6.7±5.7 | -10.7±2.0 |  | 0.217 |
|  | VDM |  | 39.4±7.2 | 39.4±7.8 | 35.4±7.6 | **<0.001** |  | 0.931 | **<0.001** | **<0.001** |  | 0.0±8.3 | -10.2±2.6 | -10.2±5.6 |  | **0.049** |
| Data are presented as mean ± standard deviation. Friedman test was used to calculate differences over three time points with a P-value (p < 0.05). Differences between two time points were calculated using the Mann-Whitney U test with a P-value (p < 0.005) (Bonferroni corrected). Wilcoxon test was used to calculate the differences (Δ) between phase 1 (T2-T1) and differences (Δ) phase 2 (T3-T2) (p < 0.05). Bold numbers indicate significant differences. CON refers to control group, VDD refers to Vitamin D daily group (800 IU daily), VDM refers to Vitamin D monthly group (50.000 IU monthly | | | | | | | | | | | | | | | | |
